# Assessment of Medial Eminence Resection Timing in Hallux Valgus Surgery Using Patient-Specific Three-Dimensional Models

**DOI:** 10.3390/jcm15135256

**Published:** 2026-07-05

**Authors:** Ahmet Atilla Abdioğlu, Göksu Yavuz Abdioğlu

**Affiliations:** 1 Department of Orthopaedics and Traumatology, Trabzon Faculty of Medicine, Trabzon University, 61030 Trabzon, Turkey; 2 Department of Pathology, Kanuni Training and Research Hospital, 61290 Trabzon, Turkey; goksu61@hotmail.com

**Keywords:** hallux valgus, distal chevron metatarsal osteotomy, medial eminence resection, metatarsal axis change angle, osteotomy contact area, three-dimensional printing

## Abstract

**Background/Objectives:** Distal chevron metatarsal osteotomy (DCMO) is one of the most commonly performed procedures for hallux valgus (HV) correction. Although medial eminence resection is routinely performed during DCMO, the effect of its timing on lateral translation, angular correction, and osteotomy contact area remains unclear. The aim of this study was to assess how the timing of medial eminence resection affects lateral translation, angular correction, and osteotomy contact area. **Methods:** Patient-specific first metatarsal models were generated from computed tomography data of 14 patients with HV using three-dimensional printing. Four groups were established according to medial eminence resection timing (before or after osteotomy) and lateral translation strategy (fixed 7 mm translation or preservation of a 5 mm contact width). DCMO was simulated in all specimens. Osteotomy contact area and the first metatarsal axis change angle (MACA) were measured and compared between the groups. **Results:** Under the fixed 7 mm translation condition, delaying medial eminence resection until after osteotomy resulted in a significantly larger osteotomy contact area than resection before osteotomy (236.50 ± 37.78 vs. 201.07 ± 22.54 mm^2^; *p* < 0.001). When lateral translation was limited by preservation of a 5 mm osteotomy contact width, delayed medial eminence resection achieved significantly greater angular correction (MACA: 20.71 ± 1.64° vs. 15.36 ± 1.45°; *p* < 0.001), while maintaining a comparable osteotomy contact area. **Conclusions:** Delaying medial eminence resection until after DCMO may allow greater lateral translation and angular correction while preserving osteotomy contact area in HV correction.

## 1. Introduction

Hallux valgus (HV), one of the most commonly encountered pathologies in foot surgery, is a complex three-dimensional deformity of the great toe [1,2]. The prevalence of HV is approximately 23% among adults aged 18–65 years and 35.7% among individuals older than 65 years [3]. The condition occurs more frequently in women than in men [3]. HV is characterized by medial deviation of the first metatarsal, lateral deviation of the hallux, and frequently accompanying pronation [1,2]. Patients with HV generally present with pain over the medially prominent bunion, and initial treatment is usually conservative [2,4]. Surgical treatment is indicated in cases of symptom progression or failure to respond to conservative management [2,4]. Achieving a satisfactory and durable correction is the primary goal of HV corrective procedures [2,5].

Contemporary surgical management of HV has undergone significant evolution, with a wide variety of corrective procedures being described, including proximal and distal metatarsal osteotomies [2,6]. The existence of more than 100 reported surgical techniques for HV correction reflects the ongoing search for an optimal procedure that can achieve reliable deformity correction while minimizing complications and recurrence rates [2,6].

The surgical technique is chosen according to the severity of the deformity as determined by radiographic measurements such as the hallux valgus angle (HVA) and the first-second intermetatarsal angle (IMA) [1,2,4,7,8]. On anteroposterior weight-bearing radiographs, normal alignment is generally defined by an HVA of less than 15° and an IMA of less than 9° [2,6,9]. Based on these radiographic parameters, HV is commonly classified as mild (HVA 15–20°, IMA 9–11°), moderate (HVA 20–40°, IMA 11–16°), and severe (HVA > 40°, IMA > 16°) [2,6,9]. Owing to their greater corrective potential, proximal metatarsal osteotomies have long been considered one of the primary surgical options for the treatment of severe HV deformities [2,6,10]. However, these procedures are associated with several drawbacks, including technical complexity, prolonged postoperative recovery, and a relatively higher risk of complications [10]. Distal metatarsal osteotomies were initially recommended for the treatment of mild to moderate HV and were traditionally considered inadequate for severe deformities because of their perceived limited corrective capacity [2,6,10]. To address these limitations, surgeons have adopted various technical modifications and adjunctive soft-tissue procedures to improve the corrective capacity of distal metatarsal osteotomies, thereby expanding their indications for the treatment of more severe HV deformities [4,5,6,11,12,13,14,15,16].

Distal chevron metatarsal osteotomy (DCMO) is a commonly used technique for the correction of HV [2,5,7,8,17]. In the classical surgical approach, the distal-medial eminence of the first metatarsal is excised prior to osteotomy to prepare the surgical field, followed by chevron osteotomy and lateral translation and fixation of the distal fragment [4,6,8,18]. Subsequently, the excess bone remaining at the distal medial aspect of the metatarsal is resected [6,8,18]. However, there is no clear consensus in the literature regarding the timing and extent of medial eminence resection [11,16,19,20,21,22].

The medial eminence is a controversial structure in both the pathogenesis and surgical management of HV [1]. Some researchers have suggested that this prominence is not a pathological bone formation, but rather a component of normal anatomy that becomes more apparent secondary to the deformity [1,23,24,25]. In this context, extensive resection of the medial eminence prior to DCMO may reduce the width of the metatarsal head, thereby limiting the lateral translation capacity of the distal fragment and potentially adversely affecting surgical stability. Indeed, Trnka and Hofstaetter emphasized that minimal resection of the medial eminence preserves metatarsal head width, allows more effective lateral translation, and represents a critical surgical principle, particularly in moderate and severe deformities [11]. It is well established that the amount of correction achieved with DCMO is limited by the lateral translation capacity of the distal fragment [15]. This further highlights the potential importance of preserving the available bone stock for surgical success. Esemenli et al. reported that the metatarsal head must be translated laterally by at least an average of 7.2 mm to achieve anatomical reduction of the sesamoids [22]. This finding supports the importance of preserving metatarsal head bone reserve in order to achieve adequate translation.

In the current literature, studies questioning the necessity of routine resection of the medial eminence prior to DCMO are limited, and the effects of preserving this structure on deformity correction have not been sufficiently clarified. The aim of this study was to comparatively evaluate the effects of the timing of medial eminence resection during DCMO on the amount of deformity correction and the contact surface area between osteotomy fragments. For this purpose, a quantitative analysis of surgical technique variations was performed using three-dimensional models derived from patient-specific computed tomography data.

## 2. Materials and Methods

### 2.1. Study Design and Patient Selection

This experimental study received approval from the local ethics committee. Informed consent was obtained from all participating patients. No new tests or examinations were performed on the patients. Only hospital records were used. The hospital information system was utilized to identify patients aged between 20 and 50 years who had undergone foot computed tomography for any indication between 1 January 2024 and 1 January 2025. The medical records of these patients were retrospectively reviewed, and cases with weight-bearing foot radiographs obtained due to HV-related complaints were evaluated. Patients with HVA > 15° and first-second IMA > 9° were accepted as HV and included in the study [2]. Patients with additional pathologies affecting the first metatarsal were excluded. Based on the predefined inclusion and exclusion criteria, a total of 14 patients were enrolled in the study, including 8 patients with moderate HV and 6 patients with severe HV.

### 2.2. Three-Dimensional Modeling and Production Process

During the creation of the three-dimensional models, computed tomography data of the patients were obtained in DICOM format, and segmentation was performed to isolate the first metatarsal bone from the surrounding tissues. During the segmentation process, bone tissue was identified using density-based thresholding methods, and anatomical accuracy was ensured through semi-automatic and manual corrections when necessary. The obtained segments were converted into three-dimensional surface models, exported in STL format, and used in the model production process. Each metatarsal model was duplicated into four copies to enable comparative analysis.

The production process was performed using a three-dimensional printer with fused deposition modeling (FDM) technology (CREALİTY K1 Max, FCC ID: 2AXH6-K1MAX, Shenzen Creality 3D Technology Co., Ltd., Shenzhen, China). All models were printed with Hyper PLA White filament (CREALITY HYPER SERIES 3D PRINTER FILAMENT) at 100% infill density. This approach aimed to maximize mechanical integrity and inter-sample standardization. A total of 56 metatarsal models belonging to 14 patients (four copies from each patient) were obtained. Models belonging to the same patient were assigned to different experimental groups to allow intra-patient comparison.

### 2.3. Experimental Groups and Surgical Simulation

**Figure 1:**
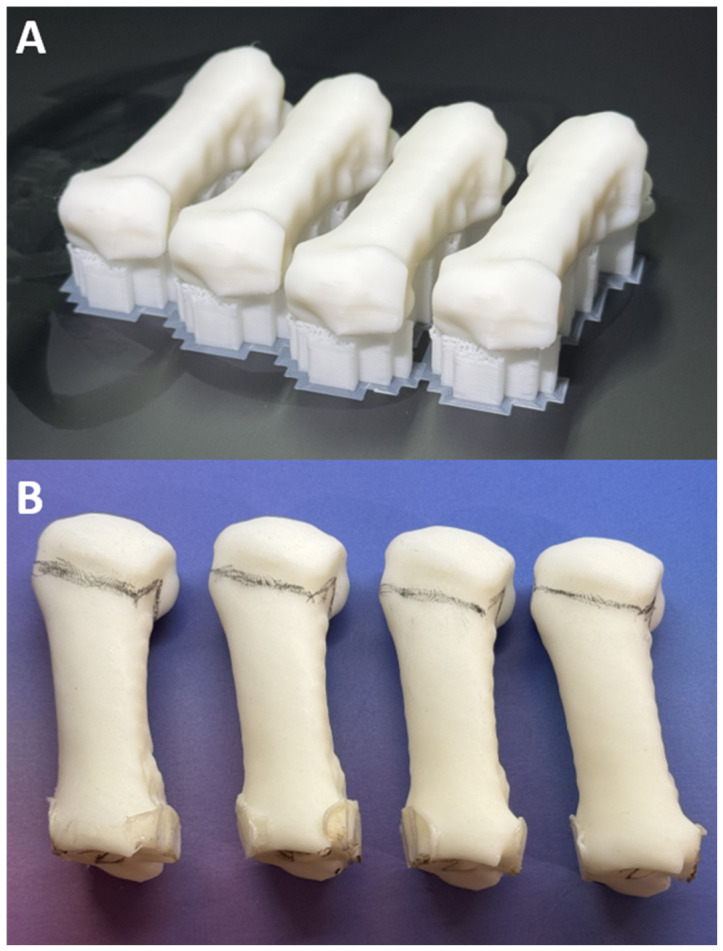
Four copies of the same metatarsal seen with support elements on a three-dimensional printer bed (**A)**. Image of identical osteotomy lines drawn from the same guide points (**B**).

All models were divided into four groups, with one model from each patient allocated to each group. DCMO was simulated in all specimens [5,26,27]. Osteotomy lines were marked in advance with a pencil in order to ensure standardization across all groups (Figure 1).

The medial eminence resection boundary was determined based on the portion remaining medial to an imaginary line extending along the medial diaphyseal cortex of the first metatarsal [1,6]. Based on previous reports describing DCMO and its displacement limits, a lateral translation of approximately 7 mm was selected as the reference value for the control group [14,15,22]. Considering studies investigating the maximum displacement limits, surgical planning was performed to preserve a 5 mm contact width in order to maintain osteotomy stability [10,14,15,16,28]. Different variations consistent with surgical practice were applied in each group:

Group 1: The medial eminence was excised before osteotomy. Osteotomy was then performed. The distal fragment was translated laterally by 7 mm. The osteotomy fragments were stabilized with Kirschner wires. The residual medial bony prominence was subsequently excised.

Group 2: The medial eminence was excised before osteotomy. Osteotomy was then performed. The distal fragment was translated laterally while preserving a 5 mm osteotomy contact width. The osteotomy fragments were stabilized with Kirschner wires. The residual medial bony prominence was subsequently excised.

Group 3: Osteotomy was performed without prior medial eminence resection. The distal fragment was then translated laterally by 7 mm. The osteotomy fragments were stabilized with Kirschner wires. Medial eminence resection was then performed.

Group 4: Osteotomy was performed without prior medial eminence resection. The distal fragment was then translated laterally while preserving a 5 mm osteotomy contact width. The osteotomy fragments were stabilized with Kirschner wires. Medial eminence resection was then performed.

**Figure 2:**
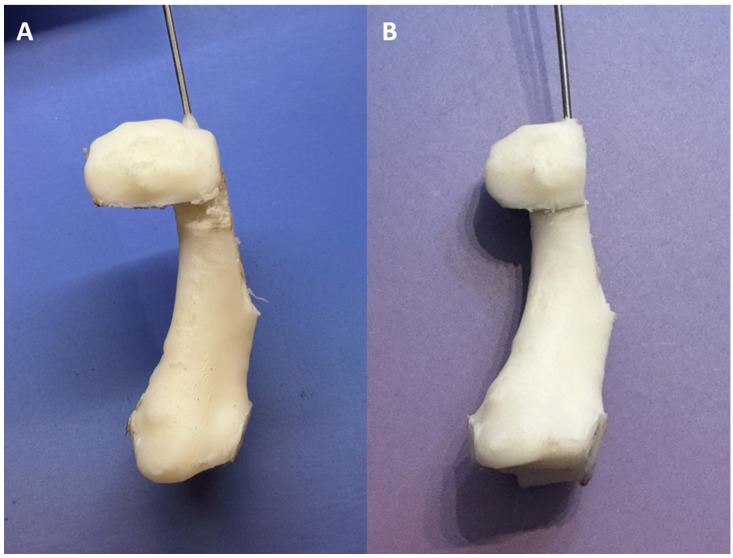
Representative postoperative images of the groups in which a 5 mm osteotomy contact width was preserved. (**A**) Osteotomy performed before medial eminence resection (Group 4), with the medial eminence resected after lateral translation. (**B**) Osteotomy performed after medial eminence resection (Group 2).

Representative images of the surgical procedures in the groups with a preserved 5 mm osteotomy contact width are shown in Figure 2.

### 2.4. Measurements and Evaluation Parameters

**Figure 3:**
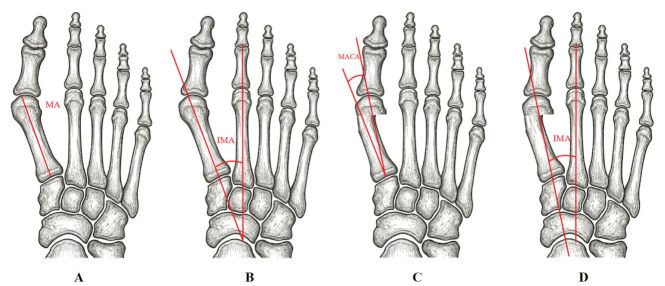
Schematic representation of the angular measurement techniques used in the study. (**A**) Determination of the first metatarsal axis (MA); (**B**) measurement of the intermetatarsal angle (IMA); (**C**) assessment of the metatarsal axis change angle (MACA) following the procedure; and (**D**) measurement of the intermetatarsal angle (IMA) following the procedure.

In each model, the change in the anatomical axis of the first metatarsal before and after the procedure was measured goniometrically and recorded as metatarsal axis change angle (MACA). All measurements were performed three times by the same experienced surgeon, and the mean value was used for the final analysis. The measurement methods for IMA and MACA are illustrated in Figure 3.

Following the angular measurements, the contact margins between the osteotomy fragments were identified and marked. After removal of the K-wires, the osteotomy contact area was assessed using a manual grid-based planimetric method. The contact surface was transferred onto a millimeter grid sheet routinely used in pathology practice for area estimation, and the enclosed area was calculated in square millimeters (mm^2^). All measurements were performed manually by an experienced pathologist on three separate occasions, and the mean value of the three measurements was used for the final analysis. For standardization, all measurements were rounded to the nearest whole number. Values with decimal fractions of 0.5 or greater were rounded up, whereas values with decimal fractions less than 0.5 were rounded down.

Comparative analyses were performed between Group 1 and Group 3 (effect of medial eminence resection under the condition of fixed 7 mm translation), and between Group 2 and Group 4 (effect of medial eminence resection under the condition of limited contact preservation).

### 2.5. Statistical Analysis

IBM SPSS Statistics 27.0 (IBM Corp., Armonk, NY, USA) was used for the statistical evaluation of data. Descriptive statistics for continuous variables were presented as mean ± standard deviation, while categorical variables were expressed as frequency and percentage.

Intraobserver reliability was evaluated using the intraclass correlation coefficient (ICC). All measurements were performed three times by the same observer at different time points. the mean values were used for subsequent analyses. Therefore, reliability was assessed using a two-way mixed-effects model with absolute agreement for average measurements (ICC[3,3]). Reliability analyses were performed separately for four measurement categories: contact area in the 7 mm translation group, MACA in the 7 mm translation group, contact area in the 5 mm contact line group, and MACA in the 5 mm contact line group. ICC values were interpreted according to the criteria proposed by Koo and Li, where values < 0.50 indicate poor, 0.50–0.75 moderate, 0.75–0.90 good, and >0.90 excellent reliability [29].

Since four duplicate models were created from each metatarsal, measurements from the four groups were considered to belong to the same bone, and analyses were performed using a paired measurement model based on individual metatarsals. Within this framework, normality assessment of group differences was performed using the Shapiro–Wilk test based on the distribution of differences between the two groups. When the distribution was considered to be normal, the paired Student’s *t*-test was used; otherwise, the Wilcoxon signed-rank test was preferred.

Two main comparisons were performed: (i) Group 1 versus Group 3 under the condition of fixed 7 mm lateral translation, and (ii) Group 2 versus Group 4 under the condition of limited translation with preservation of a 5 mm contact line. Effect size was calculated using Cohen’s dz coefficient appropriate for paired samples (mean difference divided by the standard deviation of the difference distribution). Statistical significance was set at *p* < 0.05, and a 95% confidence interval was used.

Sample size was determined by an a priori power analysis. Using G*Power version 3.1.9.7 software with a paired-samples *t*-test model, α = 0.05, 1 − β = 0.80, and an assumed moderate-to-large effect size based on the literature and pilot data, at least 13 metatarsals were estimated to be sufficient. The inclusion of 14 metatarsals in the present study exceeded this threshold. In addition, post hoc power analysis based on the obtained data demonstrated that the statistical power of the study exceeded 99%, particularly due to the large observed effect size for axis angle change (dz > 6).

## 3. Results

Contact area (mm^2^) and first metatarsal axis angle change (°) were measured in each of the 56 models produced from a total of 14 metatarsals. Measurements from the four groups corresponding to each metatarsal are presented separately in Table 1A,B.

### 3.1. Comparison Under 7 mm Translation (Group 1 vs. Group 3)

In the models in which medial eminence resection was performed before osteotomy (Group 1), the mean contact area was 201.07 ± 22.54 mm^2^, whereas this value increased to 236.50 ± 37.78 mm^2^ in the models in which medial eminence resection was performed after osteotomy (Group 3). Paired *t*-test analysis demonstrated a highly significant difference between the two groups (*p* < 0.001; Cohen’s dz = 1.62).

In terms of axis angle change, the mean values were measured as 14.00 ± 1.18° for Group 1 and 13.43 ± 1.28° for Group 3, respectively. Since the distribution of differences for this variable did not satisfy the assumption of normality (Shapiro–Wilk *p* = 0.032), the Wilcoxon signed-rank test was applied, revealing a statistically significant difference between the two groups (*p* = 0.021; Cohen dz = 0.76).

### 3.2. Comparison Under Limited Contact (5 mm) (Group 2 vs. Group 4)

The mean contact areas were 165.79 ± 17.66 mm^2^ in Group 2 and 164.07 ± 18.12 mm^2^ in Group 4, with no statistically significant difference detected by paired *t*-test analysis (*p* = 0.052; Cohen dz = 0.58).

In contrast, a dramatic difference was observed in the axis angle change. The mean value was 15.36 ± 1.45° in Group 2 and 20.71 ± 1.64° in Group 4, respectively. Paired *t*-test analysis demonstrated that this difference was highly statistically significant and associated with a very large effect size (*p* < 0.001; Cohen’s dz = 6.36).

**Figure 4:**
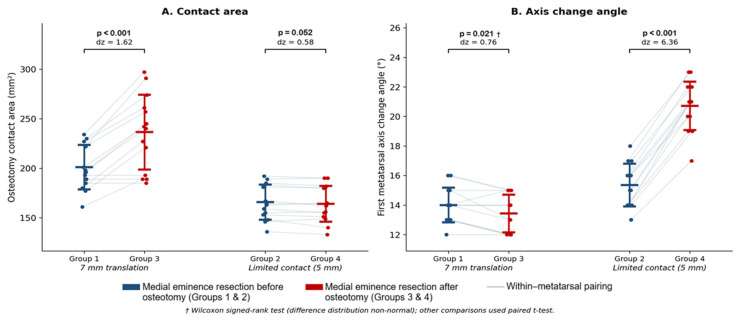
Visual representation of paired measurements. Panel A shows osteotomy contact area (mm^2^), and Panel B shows first metatarsal axis change angle (°). Each thin gray line connects the two paired measurements obtained from a single metatarsal across the two conditions; the bold horizontal markers represent group means, and the vertical error bars indicate ±1 standard deviation. The left side of each panel compares the 7 mm fixed translation condition (Group 1 vs. Group 3), and the right side compares the limited translation condition with 5 mm contact preservation (Group 2 vs. Group 4). The large and within-subject consistent difference in axis change angle between Group 2 and Group 4 is particularly notable.

A summary of all comparisons is presented in Table 2, and visualization reflecting the paired structure of the observations is shown in Figure 4.

The ICC values for osteotomy contact area measurements were 0.991 and 0.972 for the 7 mm translation and 5 mm contact line groups, respectively, indicating excellent intraobserver reliability. Angular measurements also showed high reliability; the ICC values were 0.893 and 0.966, respectively, demonstrating good and excellent intraobserver reliability.

## 4. Discussion

The principal finding of this study is that greater lateral translation can be achieved in DCMO while preserving a similar osteotomy contact area when medial eminence resection is delayed until after osteotomy. This approach may not only allow more effective correction of the existing deformity but may also increase the applicability of the same surgical technique in more advanced HV deformities, in which distal osteotomies have traditionally been considered limited.

One of the major advantages of distal metatarsal osteotomies is the ability to allow early weight-bearing [10]. Early mobilization is clinically important because it shortens the recovery period and reduces morbidity and the risk of deep vein thrombosis [10]. However, due to the short lever arm of distal osteotomies, their correction capacity may remain limited in high-grade deformities [10]. Although shifting the osteotomy level proximally allows correction of larger deformities, Trnka and Hofstaetter reported that DCMO is a reliable option in mild, moderate, and even severe HV deformities in cases with a wide metatarsal head [11]. In this context, the technical variation described in our study appears to have the potential to increase correction capacity while preserving the biomechanical and clinical advantages of distal osteotomies.

The pathogenesis of the medial eminence remains controversial in the literature [1,23,30,31]. While some authors have suggested that this structure develops as a result of new bone formation, other studies have argued that the medial prominence actually represents a segment of the first metatarsal that becomes more exposed secondary to medial deviation of the proximal phalanx [24,25,30,31]. Indeed, studies by Thordarson and Krewer, as well as Coughlin and Jones, demonstrated that medial eminence dimensions are similar in individuals with and without HV deformity, indicating that bone proliferation is not a fundamental component of bunion pathology [1,23]. In light of these findings, preservation of the medial eminence does not appear to create a disadvantage in terms of bone quality or biological reserve. On the contrary, it may contribute to the osteotomy contact surface. Although delaying medial eminence resection until after osteotomy created a slight disadvantage in IMA correction (*p* = 0.021), our study demonstrated that it provided a significant contribution to the contact area (*p* < 0.001).

Adequate lateral translation of the distal fragment is critically important for successful HV correction in DCMO [6,13,15,32]. Classical studies have recommended lateralization of the distal fragment by approximately 25–50% of the metatarsal width. However, this amount may be insufficient in cases with high IMA values [10,26,27,28]. The maximum amount of lateral translation reported in the literature varies considerably, ranging from an average of 5–7 mm to 8–10 mm, while some percutaneous techniques have described translations reaching up to 80% of the metatarsal head [4,6,14,15,28,33]. Increased translation provides stronger deformity correction, but it is known to increase the risk of complications if adequate contact surface and stability are not preserved [15]. Therefore, the balance between translation amount and osteotomy contact area plays a decisive role in surgical success.

Badwey et al. reported in a cadaveric study that the lower limit of first metatarsal head width was 11.3 mm [28]. According to traditional surgical principles, preservation of at least 50% osseous contact across the osteotomy surfaces is recommended to maintain osteotomy stability and promote reliable union [28]. Based on these anatomical considerations, the maximum lateral translation required to displace the distal fragment by approximately 50% of the metatarsal head width was reported to be 6 mm in males and 5 mm in females [28]. Nevertheless, several modified techniques have demonstrated that greater degrees of translation can be achieved safely [15,20,33]. On the other hand, the minimum lateral translation required to achieve complete reduction of the sesamoids beneath the metatarsal head has been reported to be 7.2 mm [22]. In light of these findings, 7 mm translation used in the present study represents a functional threshold for sesamoid reduction and deformity correction, whereas 5 mm contact distance represents a safe lower limit for maintaining osteotomy stability and union potential.

The amount of IMA correction achieved following DCMO has been reported across a wide range in the literature. Kyung et al. reported a mean IMA correction of 7.9 ± 3.2°, while Su et al. obtained corrections of 6.93° and 9.97° in moderate and severe deformities, respectively [4,6]. Murawski and Beskin reported a mean IMA correction of 7.9° [15]. In the percutaneous DCMO series of Vernois et al., the mean IMA correction was approximately 9° [33]. In the study by Lewis et al., which evaluated minimally invasive extracapsular DCMO, the mean IMA correction was reported as 9.6°, and it was specifically stated that medial eminence resection was not performed prior to osteotomy, while medial bony prominences were removed after displacement [16]. In contrast, some studies have reported more limited corrections, such as Kocazeybek et al., who reported a mean IMA correction of 3.71° [18]. These differences demonstrate the determining role of parameters such as surgical technique, amount of lateral translation, and osteotomy contact area on surgical outcomes. In our study, when the medial eminence was not excised before osteotomy, greater lateral translation could be achieved while preserving a similar contact area, resulting in significantly greater axial correction (*p* < 0.001). Due to the experimental design of the present study, deformity correction was assessed using MACA rather than IMA, which is more commonly reported in the literature. Consequently, direct comparison of our findings with previously published IMA-based correction values is not possible. Nevertheless, the significant differences observed in MACA measurements between the study groups provide valuable insight into the relative corrective capacities of the investigated techniques and allow meaningful comparison of their performance. This finding indicates an important technical modification that may increase the effectiveness of distal osteotomies, particularly in patients with high IMA values.

Distal chevron metatarsal osteotomy has traditionally been regarded as a procedure primarily indicated for the treatment of mild HV deformities [11,34]. In its original form, the technique was performed without internal fixation, raising concerns regarding osteotomy stability and maintenance of correction [11]. These concerns became even more relevant as the importance of lateral soft-tissue release for achieving adequate correction in more severe deformities became increasingly recognized [11]. Nevertheless, subsequent studies have demonstrated that, with appropriate patient selection and modern fixation techniques, DCMO can provide satisfactory outcomes not only in mild deformities but also in moderate and severe HV cases [11]. The characteristic V-shaped geometry of the chevron osteotomy provides an inherent mechanical advantage by contributing to the stability of the distal fragment following lateral translation [10]. However, assessment of the corrective potential of the procedure should not be based solely on osseous geometry. Soft-tissue structures, including the lateral capsule, adductor hallucis tendon, and sesamoid complex, may impose practical limitations on the amount of translation that can be achieved intraoperatively [35]. Consequently, the theoretical maximum translation values reported in the literature may not always be fully attainable under clinical conditions [28]. Furthermore, the intrinsic stability provided by the osteotomy geometry may not be sufficient in all circumstances [10]. Particularly in cases requiring substantial lateral translation, the reduction in osseous contact area may increase displacement forces across the osteotomy site, thereby amplifying the importance of stable fixation [10]. Under dynamic loading conditions, inadequate fixation or incomplete soft-tissue balancing may compromise correction and contribute to loss of alignment or malunion [10]. In addition, the foot functions biomechanically as an integrated truss system, with plantar fascial structures contributing to load transmission and stability [10]. This observation highlights that surgical success depends not only on osseous realignment but also on preservation of functional soft-tissue support mechanisms [10,25,35]. For these reasons, contemporary surgical strategies increasingly rely on fixation methods designed to enhance osteotomy stability. As more aggressive lateral soft-tissue release techniques were introduced, temporary K-wire fixation was initially adopted to reduce the risk of postoperative fragment displacement [11,15]. Moreover, fixation with two K-wires has been reported to provide significantly greater sagittal plane stability than fixation with a single K-wire [15]. Subsequently, more robust fixation methods, including compression screws, were implemented to improve construct stability [11]. In cases requiring lateral translation exceeding 50% of the metatarsal head width, the reduction in contact area may further compromise stability, making dual-screw fixation particularly advantageous [33]. Screw fixation systems used in third-generation minimally invasive techniques have been shown to provide greater stability and facilitate the safe application of larger translation magnitudes [16,33]. However, successful clinical application of such increased translation capacities requires maintenance of adequate osseous contact and the use of reliable fixation methods. Consistent with previous reports, DCMO may be safely applied in deformities with IMA of up to 18–19° when metatarsal head morphology is favorable and stable screw fixation is achieved [11].

Another methodological consideration concerns the interpretation of the effect size observed for the MACA in the comparison between Groups 2 and 4 (Cohen’s dz = 6.36). This unusually large value is largely attributable to the paired experimental design, in which each metatarsal served as its own control through physical replication. By minimizing inter-specimen anatomical variability, the design resulted in a relatively small standard deviation of the paired differences (SDdifference = 0.84°), thereby increasing the calculated dz value. The paired differences were highly consistent across all specimens (range, 4–7°), and the narrow 95% confidence interval for the mean difference [4.87°, 5.84°] further supports the precision of this finding. Therefore, the reported dz value should be interpreted within the context of the paired study design rather than as a direct indicator of clinical magnitude.

### Limitations

This study has several limitations. First, the measured angular parameter does not represent the classically defined IMA, but rather reflects axial change in the first metatarsal. This methodological preference may limit direct one-to-one comparison of the results with IMA values reported in the literature. Nevertheless, since all measurements were performed using the same technique under standardized conditions, the relative validity of intra-group and inter-group comparisons was preserved. Furthermore, the analyses were performed only on isolated first metatarsal bones and therefore do not reflect the effects of in vivo biomechanical factors such as soft tissue constraints, capsuloligamentous balance, and muscle forces. Furthermore, the three-dimensional printed models used in this study cannot completely reproduce the structural and biomechanical characteristics of native bone tissue. Accordingly, the obtained results should not be interpreted as an exact simulation of the clinical condition, but rather as a comparative evaluation of osteotomy geometry and bone contact relationships. However, since the primary aim of the study was to demonstrate the relative biomechanical effects of different techniques, the model used is considered to reliably reflect this comparison.

## 5. Conclusions

The present study demonstrated that delaying medial eminence resection until after distal chevron metatarsal osteotomy may allow greater lateral translation and consequently greater angular correction while preserving the osteotomy contact area. These findings suggest that the correction capacity of distal osteotomies may be increased through optimization of medial eminence resection timing. In addition, preserving the medial eminence until after osteotomy may provide biomechanical advantages by maintaining a larger osteotomy contact surface. Nevertheless, further studies are required to evaluate the clinical and long-term implications of these findings.

## Figures and Tables

**Table 1:** (**A**) Osteotomy contact area measurements (mm^2^) of the models. (**B**) First metatarsal axis angle change measurements (°) of the models.

**(A)**				
	**Medial Eminence Resection** **Before Osteotomy**		**Medial Eminence Resection** **After Osteotomy**	
**Metatarsal**	**Group 1** **(7 mm Translation)**	**Group 2** **(5 mm Contact)**	**Group 3** **(7 mm Translation)**	**Group 4** **(5 mm Contact)**
1	177	146	221	149
2	234	192	297	190
3	196	167	245	162
4	222	181	257	180
5	161	136	189	133
6	230	185	274	182
7	198	163	240	165
8	222	183	291	180
9	180	148	227	140
10	227	189	261	190
11	201	164	242	164
12	189	159	189	155
13	193	155	193	156
14	185	153	185	151
Mean ± SD	201.07 ± 22.54	165.79 ± 17.66	236.50 ± 37.78	164.07 ± 18.12
**(B)**				
	**Medial** **Eminence** **Resection** **Before osteotomy**		**Medial** **Eminence** **Resection** **After** **Osteotomy**	
**Metatarsal**	**Group 1** **(7 mm translation)**	**Group 2** **(5 mm contact)**	**Group 3** **(7 mm translation)**	**Group 4** **(5 mm contact)**
1	13	14	12	21
2	16	17	15	23
3	14	15	14	21
4	14	16	13	22
5	12	13	12	17
6	16	18	15	23
7	14	16	14	21
8	15	16	13	21
9	13	14	12	19
10	15	17	15	22
11	14	15	15	21
12	13	14	12	19
13	14	16	14	20
14	13	14	12	20
Mean ± SD	14.00 ± 1.18	15.36 ± 1.45	13.43 ± 1.28	20.71 ± 1.64

**Table 2:** Statistical summary of group comparisons.

	Variable	Medial Eminence Resection Before Osteotomy (B) (Mean ± SD)	Medial Eminence Resection After Osteotomy (A) (Mean ± SD)	95% CI (A–B)	*p* Value	Effect Size (dz)
Group 1 vs. Group 3	Contact area (mm^2^)	201.07 ± 22.54	236.50 ± 37.78	[22.77, 48.09]	<0.001	1.62
Group 1 vs. Group 3	Axis angle change (°) †	14.00 ± 1.18	13.43 ± 1.28	[−1.01, −0.13]	0.021	0.76
Group 2 vs. Group 4	Contact area (mm^2^)	165.79 ± 17.66	164.07 ± 18.12	[−3.43, 0.00]	0.052	0.58
Group 2 vs. Group 4	Axis angle change (°)	15.36 ± 1.45	20.71 ± 1.64	[4.87, 5.84]	<0.001	6.36

† Since the distribution of differences in this comparison did not satisfy the assumption of normality according to the Shapiro–Wilk test (*p* = 0.032), the Wilcoxon signed-rank test was used. All other comparisons were analyzed using the paired Student’s *t*-test. CI, confidence interval; SD, standard deviation; dz, Cohen’s dz effect size.

## Data Availability

The data presented in this study are available on request from the corresponding author.

## Abbreviations

The following abbreviations are used in this manuscript:

HV	Hallux valgus
HVA	Hallux valgus angle
IMA	First-second intermetatarsal angle
DCMO	Distal chevron metatarsal osteotomy
MACA	Metatarsal axis change angle
